# Effects of Non-Immersive Virtual Reality and Video Games on Walking Speed in Parkinson Disease: A Systematic Review and Meta-Analysis

**DOI:** 10.3390/jcm11226610

**Published:** 2022-11-08

**Authors:** Francisco Navarro-Lozano, Pawel Kiper, Cristina Carmona-Pérez, Sebastian Rutkowski, Elena Pinero-Pinto, Carlos Luque-Moreno

**Affiliations:** 1 Faculty of Nursing and Physiotherapy, University of Cadiz, 11009 Cadiz, Spain; 2 Laboratory of Healthcare Innovation Technology, IRCCS San Camillo Hospital, 30126 Venice, Italy; 3 Department of Nursing, Pharmacology and Physiotherapy, University of Córdoba, 14004 Cordoba, Spain; 4 Centro de Recuperación Neurológica de Córdoba (CEDANE), 14005 Cordoba, Spain; 5 Department of Physical Education and Physiotherapy, Opole University of Technology, 45-758 Opole, Poland; 6 Departamento de Fisioterapia, Universidad de Sevilla, 41009 Seville, Spain; 7 Instituto de Biomedicina de Sevilla (IBIS), 41013 Seville, Spain

**Keywords:** Parkinson disease, gait disorders, neurologic, virtual reality, exergames, quality of life

## Abstract

People with Parkinson disease suffer from a loss of dopaminergic neurons, which are involved in walking speed. Currently, virtual reality (VR) has emerged as a useful tool for the rehabilitation of people with neurological diseases, optimizing results in balance and gait. This review aimed to evaluate the effectiveness of VR or video games (through face-to-face sessions and not telerehabilitation) in improving walking speed and other spatio-temporal parameters of gait, balance, and quality of life in patients with Parkinson disease. A bibliographic search was carried out in the MEDLINE, Web of Science, Scopus, and PEDro databases. This systematic review adhered to the PRISMA guideline statement and was registered in PROSPERO (CRD42020180836). From a total of 119 records, 5 studies met the inclusion criteria for qualitative analysis, of which 3 contributed to the meta-analysis; inconclusive findings were found on gait speed, balance, and quality of life after the use of non-immersive VR systems face-to-face. A greater number of studies are necessary, with a greater number of participants, to differentiate between those VR specific systems (specifically designed for rehabilitation) from commercial video games, including immersive systems, and obtain more conclusive evidence. Furthermore, it would be interesting to compare the administration of this treatment in person versus its administration via telerehabilitation, which will help plan treatment programs.

## 1. Introduction

Parkinson disease (PD) is a neurodegenerative disease, the second most common worldwide after Alzheimer’s disease [1]. The conjugation of symptoms such as bradykinesia and postural instability generates new gait patterns in these patients characterized by a decrease in walking speed and stride length in addition to a marked trunk flexion [2,3]. Furthermore, they often present a typical disorder called "freezing", showing difficulty when starting to walk or blocking during its performance [4]. Gait hypokinesia, measured by gait speed, is correlated with the limitations on activities of daily living in people with PD (pwPD) [5], causing a sharp decrease in functionality of these patients, which can trigger an increased risk of falls, with an estimate of around 60% of pwPD falling each year [6]. Injuries caused by these falls imply a loss of mobility and autonomy in patients, with the consequent reduction in their quality of life (QoL) and an increase in mortality [6,7].

In recent decades, sensory cueing have become a powerful tool in rehabilitation to improving gait in pwPD [8]. One of the most reported interventions is the use of therapeutic exercise, including the use of external rhythms to improve gait and gait-related activities [9]. In addition to conventional techniques [10], other complementary therapies such as dance or innovative techniques including virtual reality (VR) and exergaming, motor imagery, and robotics have been emerging [11]. VR offers additional motivation to the patient, which translates into greater adherence to treatment [12].

VR technology allows the user to interact with a simulated real-time environment [13] and has the potential to improve balance and gait in neurological patients by providing additional benefits when combined with conventional rehabilitation [14,15]. The use of this type of tool has advantages over other therapies. Firstly, it provides multisensory feedback to the patient [14], combining visual and auditory stimuli. This type of sensory signal uses alternative pathways to the basal ganglia [16] to reach the premotor cortex and the supplementary motor area [17]; thus, it can compensate for the internal stimuli deficit that these patients suffer [18] to help them achieve greater control over their motor activity. Secondly, the advantages related to the different degrees of immersion that these devices allow. Immersion refers to the ability of some devices to make people physically perceive themselves in the virtual world [19,20] and is related to the degree to which the virtual reality system successfully provides an environment that refocuses patient sensations from the real world to a virtual world [21]. Concerning the degree of immersion, VR systems range from immersive to semi-immersive or non-immersive depending on the level of perceived isolation that a patient feels from the real environment when interacting with the virtual environment [22]. One of the unwanted effects that has been detected with the use of immersive systems is cybersickness (although current generation VR devices cause significantly less, some symptoms remain as intense [23], especially in patients with neurological disorders [24]). VR systems can be classified into two types according to the purpose of their hardware: specific-rehabilitation VR systems, and commercial VR video game consoles. Therefore, despite the technical and theoretical differences between immersive and non-immersive virtual reality, both technologies can have different potential impacts on the implementation of new rehabilitation treatments and allow scientists to optimize and customize experimental setups according to the needs of the patient and the hospital, including the possibility of developing telerehabilitation applications that patients can perform at home [25]. However, the differences between the possible effects derived from face-to-face applications and telerehabilitation have not been addressed previously; moreover, these particularities of face-to-face attendance could be essential for choosing the best therapeutic options according to the patient’s condition, in order to individualize patient profiles that would benefit from face-to-face rehabilitation, even if the costs are higher.

Several reviews that determine some of the benefits of the use of VR in pwPD have been reported, analyzing the improvements produced in variables such as balance [26,27] and others focusing on the effects produced by STP on gait in pwPD [28,29]. Lei et al. [28] and Dockx et al. [29], evaluated the effects of STP on gait and addressed the relationships with other types of non-motor variables, such as cognitive alterations and neuropsychiatric symptoms. Moreover, Dockx et al. [29] attempted to cover a broad field, including interventions with VR performed at the patient´s home through telerehabilitation. Unlike the previous studies, this review focuses on the effects on the STP of gait, mainly speed, and its influence on QoL, excluding telerehabilitation, since, regardless of whether the intervention is VR, the face-to-face or the telerehabilitation modality can be decisive in the results obtained. Moreover, this review is particularly interested in describing the different VR systems and protocols used in the literature. This systematic review aimed to determine the available scientific evidence on the effectiveness of VR or video games (specifically in the face-to-face modality), provided alone or in addition to conventional physical therapy (CPT), in improving walking speed in pwPD and its possible relationship with balance. As a secondary objective, we proposed the description of the different types of VR, analyzing the possible differences in terms of results for the parameters previously described.

## 2. Materials and Methods

A systematic review of the literature was developed according to the recommendations of PRISMA [30] and was registered in the PROSPERO database of the International Prospective Register of Systematic Reviews (CRD42020180836).

### 2.1. Search Strategy

An exhaustive search was carried out up to June 2022 in the following databases: *MEDLINE*, *Web of Science*, *Scopus*, and *PEDro* (Table 1). Specific search strategies for each database were employed. In these searches, descriptors such as *Parkinson’s disease* (MESH), *Parkinson disease*, *virtual reality* (MESH), *feedback* (MESH), *video games* (MESH), *Kinect*, *Wii*, *gait speed*, *gait velocity*, *walking speed* (MESH), *telerehabilitation* (MESH), and *home* were used. The detailed search strategy for the MEDLINE database is shown in Supplementary Materials (Table S1).

A manual review of gray literature was performed by checking the references of the selected articles as well as Google Scholar.

### 2.2. Eligibility Criteria

The search was restricted to studies published in English or Spanish. No filters were applied for the publication date.

The PICO strategy [31] was adopted to implement the inclusion criteria: participants: people diagnosed with PD; intervention: use of VR devices or video games aimed at treating motor symptoms of PD, mainly related to gait disturbances; comparison: CPT-based interventions or complementary therapies; and outcomes. Measurements of variables were obtained from specific and validated tests, scales, or instrumented movement analysis devices that measure the STP of gait. 

Criteria for exclusion were as follows: studies that used vibratory, auditory, or visual stimuli in isolation, without being integrated into the definition of VR systems; studies that did not analyze the STP of gait and/or walking speed; studies that focused their interventions only on addressing cognitive alterations; studies carried out on subjects with other neurological pathologies (including parkinsonism); studies that specifically carried out VR through telerehabilitation; and studies without a comparison group. This last exclusion was carried out in an attempt to clarify the specific effects of face-to-face virtual reality without the telerehabilitation modality being able to influence the results.

### 2.3. Selection Process and Data Extraction

The search was carried out by combining the keywords previously described in the different databases. Potentially relevant articles were identified after reading the title and abstract, eliminating duplicate articles. Subsequently, an exhaustive verification was carried out according to the inclusion/exclusion criteria to select the articles included in this systematic review.

Two independent reviewers (FNL and CLM) were actively involved in the processes of study selection, review, and systematic extraction of data. An additional reviewer (PK) participated in reaching a consensus for articles’ inclusion. Demographic data and some specific characteristics of the intervention were collected: author, year of publication, the total number of participants, number of participants (total and in both groups), average age, functional stage, characteristics of the intervention of VR (level of immersion, type of feedback, number total of sessions, and their timing), measurement instruments and results.

### 2.4. Qualitative and Quantitative Assessment of Treatment Effects

Methodological quality and risk of bias of randomized clinical trials (RCT) were evaluated using the Critical Appraisal Skills Programme (CASP) RCT checklist [32]. To determine the level of evidence and the grade of recommendation, the Oxford Center for Evidence-Based Medicine (CEBM) classification [33] was used; its application facilitates the ranking of available evidence in studies with different methodological designs, such as in the case of this review.

We used Review Manager 5.3 (RevMan 2014) to conduct a review, record descriptive information for each study in the characteristics of the included studies tables, assess the methodological quality of trials using risk of bias tables, and for statistical analysis. Treatment effects were evaluated using mean difference for homogeneous outcome measures or standardized mean difference (SMD) for outcomes evaluated with different scales. 

The confidence interval (CI) for continuous outcomes was identified at 95%. Statistical heterogeneity was assessed with I² statistics, establishing the cut-off value at 50% while considering intervention and outcome measures. I² statistic of ≤1% describes no or minimal heterogeneity in all of our analyses. According to Higgins et al. (2003) [34], the percentage of variation (I² statistic) between the different studies is not due to hazard but to heterogeneity. We conducted a meta-analysis based on a random effect model or fixed model with 95% CI using RevMan 5.3.

## 3. Results

**Figure 1:**
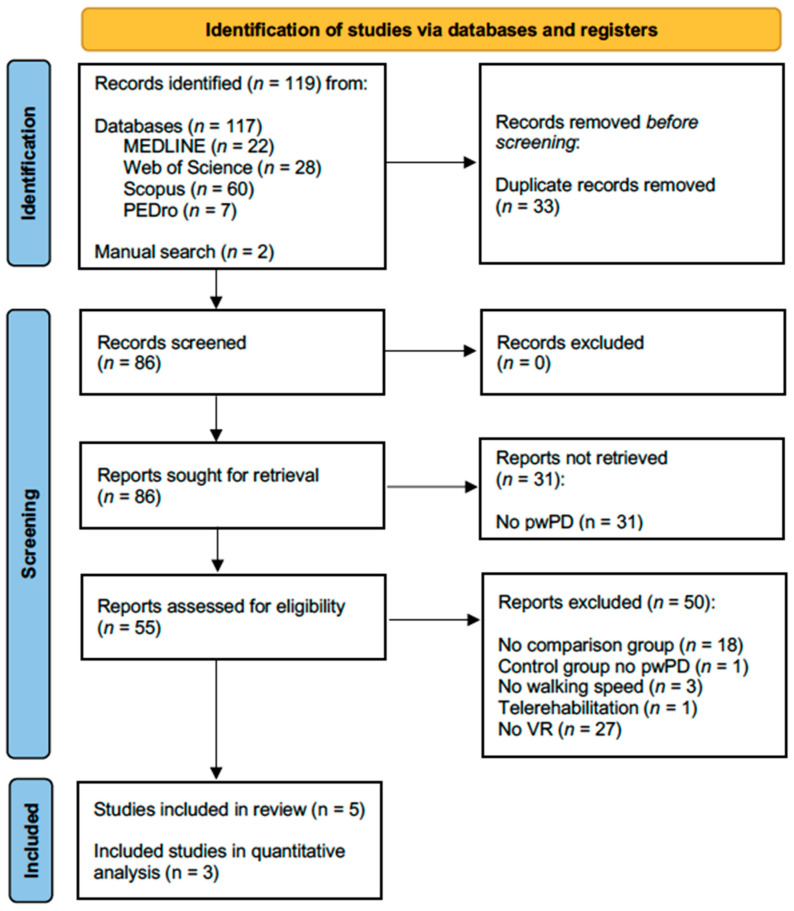
Flowchart of the selection process according to PRISMA standards [30].

After this selection process, 5 articles that met the inclusion and exclusion criteria were analyzed for this review. The different stages of the search developed are shown in Figure 1. 

Following a systematic process of searching and selecting studies, five articles met the inclusion criteria and were included in the review, including four RCTs and one non-randomized quasi-experimental clinical trial (NRS) and bringing together a total of 185 participants. A list was compiled to show the items that were excluded and the reasons for exclusion, which can be found in Supplementary Materials (List S1).

### 3.1. Assessment of Methodological Quality of the Studies

The results of the Assessment of Methodological Quality of the Studies are shown in Table 2.

### 3.2. Synthesis of Results

The synthesis of results are shown in Table 3.

### 3.3. Risk of Bias

**Figure 2:**
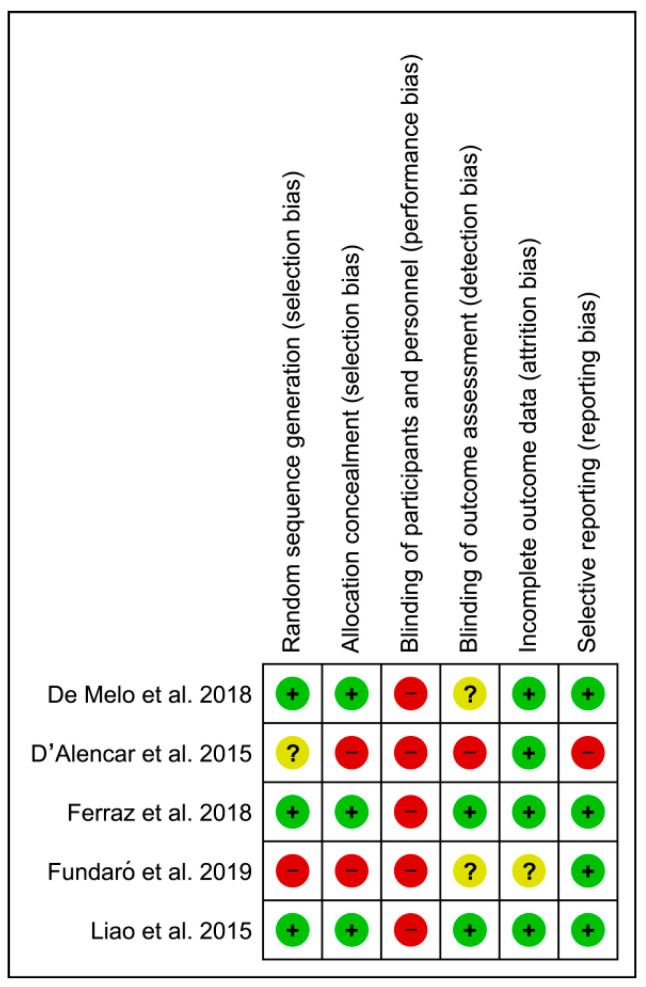
Risk of bias of the studies included in the systematic review [36,37,38,39,40].

**Figure 3:**
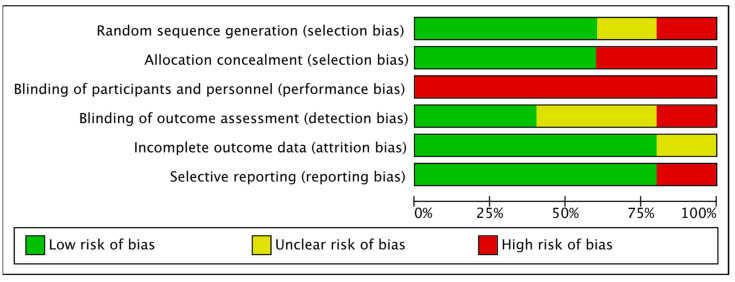
Overall risk of bias. The results are presented as percentages.

The risk of bias for the included studies was independently assessed by two reviewers, who were supported by a third researcher in case of disagreement. The assessment was carried out following the criteria stated by the Cochrane Collaboration in the Cochrane Handbook for Systematic Reviews of Interventions [35]. We evaluated the following domains: (1) selection bias: sequence generation and allocation concealment; (2) performance bias: blinding of participants and researchers; (3) detection bias: blinding of outcome assessment; (4) attrition bias: incomplete outcome data; and (5) reporting bias: selective reporting. We coded the risk of bias for each domain as ”high risk”, in cases with a high possibility of occurrence of bias; “low risk”, in cases with a low possibility of bias; and ”unclear risk”, when we could not exactly define the real incidence of bias. Figure 2 and Figure 3 show the risk of bias in the included trials. 

“Random sequence generation” (selection bias): Three studies resulted in a low risk of bias, as the authors described a random component in the sequence generation process. One study did not report information about the randomization process, resulting in an unclear risk of bias. One study resulted in a high risk of bias, with no appropriate randomization methods used.

“Allocation concealment” (selection bias): Three studies with a low risk of bias were evaluated, as the allocation methods used were appropriate. Two studies had a high risk of bias, as the therapists could anticipate the assignment of patients.

“Blinding of participants and personnel” (performance bias): All five studies presented a high risk of bias as participants or staff were aware of treatment.

“Blinding of outcome assessment” (detection bias): Two studies had a low risk of bias, as the outcome measures were evaluated by therapists that differed from those providing treatment sessions. Two studies did not state whether assessors were blinded, so the risk of bias was unclear. One study was judged to have a high risk of bias, as evaluations and treatment programs were carried out by the same therapist. 

“Incomplete outcome data” (attrition bias): Four studies had a low risk of bias since most of the participants were included in the final analysis. One study did not report outcome data.

“Selective reporting” (reporting bias): Four studies reported all the pre-specified outcomes. Only one study did not publish all outcome measures registered in the study protocol, resulting in a high risk of bias.

### 3.4. Participant Characteristics

The studies had small sample sizes ranging from 20 to 62 participants. The youngest participants were included in the study by De Melo et al. [38] and the oldest in the study by D’Alencar et al. [36].

In most studies, patients presented a score on the Hoehn and Yahr (H&Y) scale between 2 and 3, except the study by De Melo et al. [38], whose intervention group had a score below 2.

### 3.5. Intervention Characteristics

Despite some heterogeneity concerning the protocols used in the different studies, they present a mean of 14 sessions with 31 minutes of intervention using VR systems. Moreover, the dose of intervention was classified as a mean of three sessions per week.

#### 3.5.1. Commercial Systems Adapted for Therapeutic Use in Patients

- Nintendo Wii Balance Board (WBB)
  1. D’Alencar et al. [36] carried out an intervention based on seven Wii Fit virtual games with the Wii Balance Board (WBB) platform that required active movements from the participants during 35-minute sessions. The results were compared with a control group that received HR sessions of similar duration.
  2. Liao et al. [37] adapted the use of the Wii Fit Plus and the Wii Fit Balance Board to perform a 45-minute intervention protocol divided into three exercise modes (10 minutes of yoga, 20 minutes of balance, and 15 minutes of training strength), along with an additional 15 minutes of treadmill training. This group was compared with two groups. One of them received treadmill and CPT treatment for the same duration as the VR group, and the remaining group (called the control group) only received fall prevention talks.
- Kinect Xbox 360 (KX)
  1. De Melo et al. [38] chose the game “Your shape Fitness Evolved 2012”. In this video game, the patient had to perform during 20-minute sessions by simulating walking and running movements and using knee lifts without moving from his position. They also compared the effect of this intervention on two other groups. The first received CPT sessions and the second received treadmill training sessions.
  2. Ferraz et al. [39] used a 30-minute intervention with the game "Kinect Adventures" for the experimental group. The patient was asked by the avatar that appeared on the screen to perform full-body movements to achieve goals with the avatar that appeared on the screen. They compared the intervention with two additional groups, both of which performed 30-minute sessions of aerobic exercise (via cycle ergometer or functional exercises). In addition to their specific intervention, all groups performed stretching, warm-ups, and breathing exercises for 20 minutes.

#### 3.5.2. Combination of Treadmill + VR (Images on a Screen of an Avatar of Themselves)

Fundaró et al. [40] relied on the use of the Lokomat robotic gait that supports 30% of the patient’s weight, thus helping to train walking on a treadmill in 30-minute sessions. The patient’s goal was to walk on the treadmill and reach certain virtual objects that were reproduced on a screen. The results were compared with those obtained by a control group that carried out walking training on the ground. Both groups also received a 60-minute daily CPT treatment.

### 3.6. Effects of Intervention

#### 3.6.1. Comparation 1. Gait Speed

**Figure 4:**
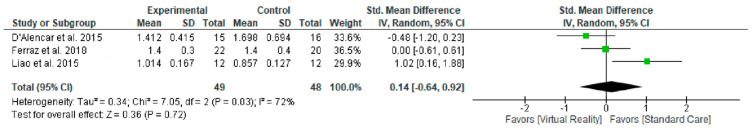
Comparison 1. Virtual reality versus standard care treatment. Outcome: gait speed. A green block indicates the weight assigned to the study, and the horizontal line depicts the confidence interval. A black rhombus shows the overall result [36,37,39].

We included three studies with a total of 97 participants. We used standardized mean differences with a random effect model since outcomes measures differed between trials. The analysis did not show differences between the groups in the RCT (SMD = −0.13; 95% CI −0.53–0.27) with I^2^ = 0% heterogeneity (Figure 4).

#### 3.6.2. Comparation 2. Balance

**Figure 5:**
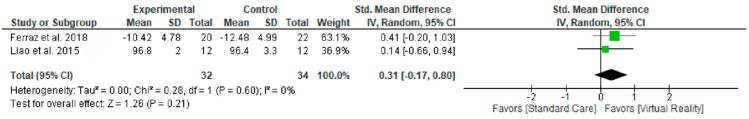
Comparison 2. Virtual reality versus standard care treatment. Outcome: balance. A green block indicates the weight assigned to the study, and the horizontal line depicts the confidence interval. A black rhombus shows the overall result [37,39].

We included two RCT studies with 66 patients overall. We used standardized mean differences with a random effect model since outcomes measures differed between trials. The analysis did not show differences between the groups in the RCT (SMD = −0.34; 95% CI −0.14–0.83) with I^2^ = 0% heterogeneity (Figure 5).

## 4. Discussion

In this review, we analyzed the specific effects of virtual reality when they are not applied to the telerehabilitation modality. Given the specificity, the number of selected studies is small, and our conclusions cannot be emphatic. However, we will detail some significant aspects that can guide future clinical trials on this topic.

Regarding the types of VR used, most studies used non-immersive VR, probably because several commercial devices can be better adapted to the needs of patients [41,42]. Another factor that could have influenced this decision is that immersive modalities have been associated with adverse events, such as anxiety [43] or cybersickness, in people with neurological disorders [42,44]. However, the current literature shows that the use of full immersion is safe for pwPD [45] in addition to the greater sense of commitment or presence that these systems provide to patients [46].

There is no clear relationship between the improvement of a patient and a specific number of sessions or hours of treatment. A special mention should be given to the achievement of significant improvements in walking speed as a short-term effect in the study carried out by Liao et al. [37]; the improvements were maintained after one month with an overall of 12 sessions for 40-minute each.

According to the analyzed studies, the use of this type of therapy is shown to be safe. Only the study by Ferraz et al. [39] showed adverse effects in two patients, who dropped out of the intervention (one due to lack of adherence and the other due to increased blood pressure). Moreover, the fact that more than 85% of patients completed the treatment shows optimal adherence. In contrast, the control group in the study by Liao et al. [37] only received fall prevention talks, and the loss of a participant due to low motivation was reported, reinforcing the important motivational factor inherent in VR interventions whose relevance has already been mentioned [47,48].

It appears that interventions with a mean of 3 weekly 30-minute sessions using visual and auditory feedback in patients with a score of less than 3 on the H&Y scale reported greater benefits. In addition to showing a high level of safety due to the low number of adverse effects detected, VR or video game interventions also reported optimal adherence, thus strengthening the important motivational factor of these interventions.

### 4.1. Walking Speed

Three of the five selected studies [37,38,39] showed significant improvements within the group in this parameter after VR interventions and Liao et al. [37] exclusively found a statistically significant difference between the groups with respect to the control group. However, the meta-analysis did not show the superiority of VR intervention over standard care. In this sense, a current Cochrane review [29] showed low-quality evidence of a positive effect of short-term VR exercise on step and stride length, defending that VR and physiotherapy may have similar effects on gait, balance, and quality of life. In our revision, the authors used a VR treatment time (40 min) higher than the mean average of the other selected studies and that, therefore, may be one of the factors that favors their improvements relative to the control group. They also used treadmill training, which can help to achieve improvements in walking speed because there is evidence of improvements in this parameter with this treatment [49]. It should also be noted that three of the studies [37,38,39] that carried out their protocols from commercial VR devices (Kinect Xbox and WBB) through the combined use of visual and auditory feedback achieved significant within-group improvements in walking speed (and inter-group in the case of Liao et al. [37]), had a higher methodological quality, and had a higher level of evidence.

Specific-rehabilitation VR systems incorporate principles of neurorehabilitation that potentially enhance learning and recovery, whereas commercial VR video game consoles are mainly designed for entertainment purposes. These benefits exposed by the use of VR and video games increase therapeutic adherence in the patient and motivate him to continue with treatment [47]. This information is important since progressive loss of dopamine in the basal ganglia in these patients generates a lack of motivation [48] and attention deficit [50], so this type of intervention may be useful to implement alone [51] or as part of an overall treatment model where it would provide an additional effect [52].

Two of the studies [38,39] showed improvements in the 6MWT test, which indicates that in addition to achieving an increase in walking speed patients were able to maintain this improvement for a longer period (greater resistance) [53] compared to the improvements evaluated by other tests, such as the 10MWT. However, the results are not conclusive for this variable in favor of any of the groups.

### 4.2. Balance and Quality of Life

Balance was analyzed in the studies by Ferraz et al. [39] and Liao et al. [37] using the sensory organization test (SOT) and sitting and rising test. In both studies, significant intragroup improvements were found after the intervention. Liao et al. [37] highlighted the significant improvements obtained in SOT in favor of the group using VR, which were maintained one month after the end of the intervention. Liao et al. carried out a specific balance treatment using the Wii Fit, which provides feedback from the pressure center [37]. However, the results are not conclusive for this variable in favor of any of the groups according to the meta-analysis. These results contrast with those obtained by Wang et al. [26], who defend the significant effects of virtual reality on balance. In some of the included studies, the application of virtual reality was carried out through telerehabilitation, which raises a question: Could it be that telerehabilitation facilitates more intensive rehabilitation that offers better results? Other reviews [27] also advocate optimization of balance and quality of life when VR is used in combination with CPT, but the values demonstrated a poor methodological quality and a low level of completeness of the intervention descriptions despite all using Nintendo Wii. Our review had as a secondary objective a more detailed description of interventions, especially for the face-to-face modality. 

Only the study by Ferraz [39] compared the quality of life of its participants using the PDQ-39 scale. It showed significant improvements in QoL within the group after its interventions, although none of the studies showed differences between groups in quantitative analysis.

### 4.3. Limitations

Some limitations have been found in the development of this review. The main ones are the small number of available studies and, in some cases, their low methodological quality. Similarly, it cannot be inferred exactly what type of intervention or what type of dosage is more appropriate due to different types of devices and intervention protocols. More randomized clinical trials with appropriate sample sizes are needed to calculate the effect size, generate more comprehensive scientific evidence, and correctly assess the specific effect of VR.

### 4.4. Clinical Implications

The clinical implications of the use of virtual reality face-to-face are the possibilities of combining it with conventional techniques that, as some authors defend, could optimize the results. Future cost-benefit studies are needed to determine whether the advantages of telerehabilitation (such as the possibility of intensifying treatment and reducing travel costs) are superior to those of face-to-face intervention. However, given the efficacy of conventional treatments, it would be important to consider the guidance of a therapist synchronously to avoid anomalous compensations, for example.

In the future, researchers should focus on comparing the effects of commercial VR systems with systems created specifically for interventions for patients because the constant technological advances in this area make great progress in these systems in short periods. Similarly, it would be interesting for future studies to include long-term follow-ups of patients to discover the maintenance over time of these interventions, and groups should be divided by H&Y stages to find out the particular effects on different types of patients. In addition, it would be interesting for future studies to compare the efficacy of face-to-face VR versus telerehabilitation.

## 5. Conclusions

The results obtained from the studies analyzed show that the use of VR or video games face-to-face could bring some advantages in improving walking speed, balance, or QoL in pwPD. However, these improvements cannot be confirmed due to the low number of studies analyzed and the small sample size.

It would be necessary to clarify, in future research, the isolated effect of VR, as well as its combined effect with conventional rehabilitation, to demonstrate whether this treatment would optimize the results of functional recovery in pwPD. Furthermore, it would be interesting to compare the administration of this treatment in person versus its administration via telerehabilitation, which will help plan treatment programs.

## Figures and Tables

**Table 1:** Search Strategy.

Database	Search Terms	Records
Web of Science	**TOPIC:** (“parkinson disease” OR “parkinson´s disease”) AND (“virtual reality” OR feedback OR “video games” OR “Kinect” OR “Wii”) AND (“gait speed” OR “gait velocity” OR “walking speed”) NOT (“Telerehabilitation" OR home*)	28
Scopus	**TITLE-ABS-KEY** (“parkinson disease” OR “parkinson´s disease") AND (“virtual reality” OR feedback OR “video games” OR “Kinect” OR “Wii”) AND (“gait speed” OR “gait velocity” OR “walking speed”) AND NOT (“Telerehabilitation” OR home*)	60
PEDro	“parkinson disease” “virtual reality” “video games”	7
Medline	(“parkinson disease” OR “parkinson´s disease”) AND (“virtual reality” OR feedback OR “video games” OR “Kinect” OR “Wii”) AND (“gait speed” OR “gait velocity” OR “walking speed”) NOT (“Telerehabilitation” OR home*)	22
Total		117

**Table 2:** Scores obtained after methodological evaluation according to the CASP Checklist [35].

Study		D’Alencar et al., 2015 [36]	Liao et al., 2015 [37]	De Melo et al., 2018 [38]	Ferraz et al., 2018 [39]	Fundarò et al., 2019 [40]
Is the basic study design valid for a randomized controlled trial?	Did the study address a clearly focused research question?	YES	YES	YES	YES	YES
	Was the assignment of participants to interventions randomized?	UNK	YES	YES	YES	NO
	Were all participants who entered the study accounted for at its conclusion?	YES	YES	YES	YES	YES
Was the study methodologically sound?	Were the participants and investigators ”blind”?	NO	NO	NO	NO	NO
	Were the study groups similar at the start of the randomized controlled trial?	NO	YES	YES	YES	YES
	Did each study group receive the same level of care?	YES	YES	YES	YES	YES
What are the results?	Were the effects of the intervention reported comprehensively?	YES	YES	YES	YES	YES
	Was the precision of the estimate of the intervention or treatment effect reported?	YES	YES	YES	YES	YES
	Do the benefits of the experimental intervention outweigh the harms and costs?	YES	YES	YES	YES	YES
Will the results help locally?	Can the results be applied to your local population/in your context?	YES	YES	YES	YES	YES
	Would the experimental intervention provide greater value to the people in your care than any of the existing interventions?	YES	YES	YES	YES	YES
Total		8/11	10/11	10/11	10/11	9/11

*UNK*: Unknown.

**Table 3:** Synthesis of results.

Author (y) LE, GR	Study Design	Age Mean	Sample	Stage * (Mean)	Levels of Immersion-Intervention-FB	Sessions	Outcome Measures	Results
D’Alencar et al. (2015) B, 2b [36]	RCT	70	IG = 15 CPTG = 16	IG = 2.2 CPTG = 2.3	- Non-immersive - IG = WBB - FB: Visual/auditory.	10 ses 35 m/ses 3 d/wk	10MWT	No statistically significant improvement in ws post-intervention. Correlation between Parkinson disease stage and ws.
Liao et al. (2015) A, 1b [37]	RCT	66	IG = 12 CPTG = 12 CG = 11	GE = 2 CPTG = 2 CG = 1.9	- Non-immersive - IG = WBB + treadmill. - FB: Visual/auditory.	12 ses 40 m/ses 2 d/wk	STP (GAITRite); FGA; Dynamometer; SOT	IG and CPTG significant improvements in stride length, speed, and FGA over CG (post one-month follow-up). No difference between IG and CPTG. SOT: Significant improvements in IG and CPTG over CG in visual (post, one-month). Improvements in vestibular in IG with respect to CG.
De Melo et al. (2018) A, 1b [38]	RCT	62	IG = 12 TG = 13 CPTG = 12	IG = 1.4 TG =1.5 CPTG = 2.08	- Non-immersive - IG = KX 360. - FB: Visual/auditory.	12 ses 20 m/ses 3 d/wk	UPDRS; 6MWT; IMU	Statistically significant increase in ws of IG and TG with respect to CPTG.
Ferraz et al. (2018) A, 1b [39]	RCT	69	IG = 20 CPTG = 22 CEG = 20	IG = 2.5 CPTG = 2.5 CEG = 2.5	- Non-immersive - IG = KX 360 + CPT - FB: Visual/auditory.	18 ses 30 m/ses 3 d/8wk	UPDRS; PDQ-39; 6MWT; 10MWT	Only IG achieved significant improvements in 10 MWT. IG also showed significant improvements in 6MWT and PDQ-39 as well as the other two groups.
Fundarò et al. (2019) B, 2b [40]	NRS	68	VRLG = 10 CPTG = 10	VRLG = 2.5 CPTG = 2.5	- Non-immersive - VRLG = Lokomat treadmill + VR + CPT. - FB: Visual/auditory.	20 ses 30 m/ses 5 d/wk	UPDRS; FIM; 10MWT; Speed in Lokomat; VR score	Only CPTG improved in 10MWT significantly without significant differences in VRLG. VRLG had significant improvements in the speed of the Lokomat treadmill and the VR score, although inversely correlated with the results of 10MWT.

* Parkinson disease stage was evaluated using the Hoehn & Yahr scale. *6MWT*: 6-minute Walk test; *10MWT*: 10 meters walking test; *CEG*: Cyclo-ergometer group; *CG*: Control group; *CPT*: Conventional Physical therapy; *CPTG*: Conventional physical therapy group; *d*: day; *FGA*: Functional gait performance; *FIM*: Functional Independence Measure; *GR*: Grade of recommendation; *IG*: Intervention group; *IMU*: Inertial measurement unit; *KX*: Kinect Xbox; *LE*: Level of Evidence; *m*: minutes; *NRS*: Non-randomized controlled trial; *PDQ-39*: Parkinson´s Disease Questionnaire; *RCT*: Randomized controlled trial; *ses*: sessions; *SOT*: sensory organization test; *TG*: Treadmill training Group; *UPDRS*: Unified Parkinson’s Disease Rating Scale; *VR*: Virtual reality; *FB*: Feedback; *VRLG*: Virtual reality and Lokomat group; *WBB*: Wii balance board; *wk*: week; *ws*: walking speed; *y*: year.

## Supplementary Materials

The following supporting information can be downloaded at: https://www.mdpi.com/article/10.3390/jcm11226610/s1, Table S1. Search strategy for MEDLINE database; List S1. Excluded articles and reasons.
