# Supplementary material for: Effects of Non-Immersive Virtual Reality and Video Games on Walking Speed in Parkinson Disease: A Systematic Review and Meta-Analysis
Source: J Clin Med. 2022 Nov 8;11(22):6610. doi: 10.3390/jcm11226610 (PMC9697190; doi:10.3390/jcm11226610)
Supplement: Supplementary file 1 [file jcm-11-06610-s001.zip › jcm-1946043-supplementary.pdf]

Journal of
Clinical Medicine

MDPI

SUPPLEMENTARY MATERIALS

Table S1. Search strategy for Medline database.

	Complete Search Strategy
#1	Search (“Parkinson disease” [Mesh] OR “Parkinson´s disease”)
#2	Search (“virtual reality” [Mesh] OR feedback [Mesh] OR “video games” [Mesh] OR “Kinect” OR “Wii”)
#3	Search (“gait speed” OR "gait velocity" OR “walking speed” [Mesh])
#4	Search ("Telerehabilitation" [Mesh] OR home*)
#5	Search (#1 AND #2 AND #3 AND #4)

No filters were used according to the study type.

List S1. Excluded articles and reasons.

RECORDS EXCLUDED BASES ON EXCLUSION CRITERIA (N = 46)

No people with Parkinson Disease (n =31)

1. Afzal MR, Oh M-K, Lee C-H, Park YS, Yoon J. A Portable Gait Asymmetry Rehabilitation System for Individuals with Stroke Using a Vibrotactile Feedback. Biomed Res Int. 2015.
2. Baram Y, Lenger R. Gait improvement in patients with cerebral palsy by visual and auditory feedback. In: 2009 Virtual Rehabilitation International Conference, VR 2009. 2009. p. 146–9.
3. Baram Y, Miller A. Auditory feedback control for improvement of gait in patients with Multiple Sclerosis. J Neurol Sci. 2007 Mar 15;254(1–2):90–4.
4. Baram Y. Virtual sensory feedback for gait improvement in neurological patients. Front Neural Circuits. 2013.
5. Boersma D, Demontiero O, Amiri ZM, Hassan S, Suarez H, Geisinger D, et al. Vitamin D status in relation to postural stability in the elderly. J Nutr Heal Aging. 2012 Mar;16(3):270–5.
6. Chou Y, Wagenaar RC, Saltzman E, Giphart JE, Young D, Davidsdottir R, et al. Effects of Optic Flow Speed and Lateral Flow Asymmetry on Locomotion in Younger and Older Adults: A Virtual Reality Study. JOURNALS Gerontol Ser B-PSYCHOLOGICAL Sci Soc Sci. 2009 Mar;64(2):222–31.
7. Dobkin BH. Wearable motion sensors to continuously measure real-world physical activities. Vol. 26, Current Opinion in Neurology. 2013. p. 602–8.
8. Eltoukhy M, Kuenze C, Oh J, Jacopetti M, Wooten S, Signorile J. Microsoft Kinect can distinguish differences in over-ground gait between older persons with and without Parkinson’s disease. Med Eng Phys. 2017 Jun; 44:1–7.
9. Gallagher R, Damodaran H, Werner WG, Powell W, Deutsch JE. Auditory and visual cueing modulate cycling speed of older adults and persons with Parkinson’s disease in a Virtual Cycling (V-Cycle) system. J Neuroeng Rehabil. 2016;13.
10. Gandolfi M, Geroin C, Picelli A, Munari D, Waldner A, Tamburin S, et al. Robot-assisted vs. sensory integration training in treating gait and balance dysfunctions in patients with multiple sclerosis: A randomized controlled trial. Front Hum Neurosci. 2014 May 22;8(MAY).
11. Hausdorff JM. Gait variability: Methods, modeling and meaning. J Neuroeng Rehabil. 2005;2.
12. Kim A, Schweighofer N, Finley JM. Locomotor skill acquisition in virtual reality shows sustained transfer to the real world. J Neuroeng Rehabil. 2019;16(1).
13. Lee HJ, Lee KE, Yi TI, Kim HY. Feedback Facility−Assisted Balance Training in a Patient With Multiple System Atrophy: A Case Presentation. PM R. 2018 May 1;10(5):555–9.
14. Major MJ, Twiste M, Kenney LPJ, Howard D. The effects of prosthetic ankle stiffness on stability of gait in people with transtibial amputation. J Rehabil Res Dev. 2016;53(6):839–52.
15. Montero-Odasso M, Almeida QJ, Bherer L, Burhan AM, Camicioli R, Doyon J, et al. Consensus on Shared Measures of Mobility and Cognition: From the Canadian Consortium on Neurodegeneration in Aging (CCNA). JOURNALS Gerontol Ser A-BIOLOGICAL Sci Med Sci. 2019 Jun;74(6):897–909.
16. Müller B, Ilg W, Giese MA, Ludolph N. Validation of enhanced kinect sensor based motion capturing for gait assessment. PLoS One. 2017;12(4).
17. Munoz B, Castano-Pino YJ, David Arango Paredes J, Navarro A. Automated gait analysis using a kinect camera and wavelets. In: 2018 IEEE 20th International Conference on e-Health Networking, Applications and Services, Healthcom 2018. 2018.
18. Phu S, Vogrin S, Al Saedi A, Duque G. Balance training using virtual reality improves balance and physical performance in older adults at high risk of falls. Clin Interv Aging. 2019;14:1567–77.

19. Plotnik M, Azrad T, Bondi M, Bahat Y, Gimmon Y, Zeilig G, et al. Self-selected gait speed - Over ground versus self-paced treadmill walking, a solution for a paradox. J Neuroeng Rehabil. 2015;12(1).
20. Plotnik M, Bartsch RP, Zeev A, Giladi N, Hausdorff JM. Effects of walking speed on asymmetry and bilateral coordination of gait. Gait Posture. 2013 Sep;38(4):864–9.
21. Powell W, Simmonds MJ. Virtual reality and musculoskeletal pain: Manipulating sensory cues to improve motor performance during walking. Cyberpsychology, Behav Soc Netw. 2014 Jun 1;17(6):390–6.
22. Pramodhyakul N, Amatachaya P, Sooknuan T, Arayawichanon P, Amatachaya S. Effects of a visuotemporal cue on walking ability of independent ambulatory subjects with spinal cord injury as compared with healthy subjects. Spinal Cord. 2014;52(3):220–4.
23. Prochazka A, Vysata O, Valis M, Tupa O, Schaetz M, Marik V. Bayesian classification and analysis of gait disorders using image and depth sensors of Microsoft Kinect. Digit Signal Process. 2015;47:169–77.
24. Rabin E, Shi P, Werner W. Gait parameter control timing with dynamic manual contact or visual cues. J Neurophysiol. 2016 Jun 1;115(6):2880–92.
25. Rawson KS, Creel P, Templin L, Horin AP, Duncan RP, Earhart GM. Freezing of Gait Boot Camp: feasibility, safety and preliminary efficacy of a community-based group intervention. Neurodegener Dis Manag. 2018 Oct;8(5):307–14.
26. Schauer M, Mauritz KH. Musical motor feedback (MMF) in walking hemiparetic stroke patients: Randomized trials of gait improvement. Clin Rehabil. 2003 Nov;17(7):713–22.
27. Stoller O, Waser M, Stammler L, Schuster C. Evaluation of robot-assisted gait training using integrated biofeedback in neurologic disorders. Gait Posture. 2012;35(4):595–600.
28. Tran H, Pathirana PN, Horne M, Power L, Szmulewicz DJ. Quantitative Evaluation of Cerebellar Ataxia Through Automated Assessment of Upper Limb Movements. IEEE Trans Neural Syst Rehabil Eng. 2019;27(5):1081–91.
29. Vadnerkar A, Figueiredo S, Mayo NE, Kearney RE. Design and Validation of a Biofeedback Device to Improve Heel-to-Toe Gait in Seniors. IEEE J Biomed Heal Informatics. 2018;22(1):140–6.
30. Vitório R, Lirani-Silva E, Barbieri FA, Raile V, Stella F, Gobbi LTB. Influence of visual feedback sampling on obstacle crossing behavior in people with Parkinson's disease. Gait Posture. 2013;38(2):330–4.
31. Wagner J, Martínez-Cancino R, Makeig S. Trial-by-trial source-resolved EEG responses to gait task challenges predict subsequent step adaptation. Neuroimage. 2019;199:691–703.

FULL-TEXT ARTICULES EXCLUDED (N= 50)

NO comparison group (n=18).

- **Case series (n=4)**

1. Badarny S, Aharon-Peretz J, Susel Z, Habib G, Baram Y. Virtual reality feedback cues for improvement of gait in patients with Parkinson's disease. Tremor Other Hyperkinet Mov (N Y). 2014; 4:225.
2. Gonçalves GB, Leite MAA, Orsini M, Pereira JS. Effects of using the nintendo wii fit plus platform in the sensorimotor training of gait disorders in Parkinson's disease. Neurol Int [Internet]. 2014 Jan 17 [cited 2020 Apr 1];6(1):5048. Available from: http://www.ncbi.nlm.nih.gov/pubmed/24744845
3. Mirelman A, Maidan I, Herman T, Deutsch JE, Giladi N, Hausdorff JM. Virtual reality for gait training: Can it induce motor learning to enhance complex walking and reduce fall risk in patients with Parkinson's disease? Journals Gerontol - Ser A Biol Sci Med Sci. 2011 Feb;66 A(2):234–40.
4. Park HS, Yoon JW, Kim J, Iseki K, Hallett M. Development of a VR-based treadmill control interface for gait assessment of patients with Parkinson's disease. In: IEEE International Conference on Rehabilitation Robotics. 2011.

- **Review studies (n=14)**

1. Abbruzzese G, Marchese R, Avanzino L, Pelosin E. Rehabilitation for Parkinson's disease: Current outlook and future challenges. Park Relat Disord. 2016;
2. Cano Porras D, Siemonsma P, Inzelberg R, Zeilig G, Plotnik M. Advantages of virtual reality in the rehabilitation of balance and gait: Systematic review. Neurology. 2018 May 29;90(22):1017–25.
3. Ferraz DD, Trippo K, Dominguez A, Santos A, Oliveira Filho J. Nintendo Wii training on postural balance and mobility rehabilitation of adults with Parkinson's disease: a systematic review. Fisioter em Mov. 2017;30(suppl 1):383–93.
4. Ford MM, Howell JB, Moore BC, St. Aimie ST, Cook LC, Weaver KR, et al. The effect of dual task activities on the walking gait of individuals with Parkinson's disease. Clin Kinesiol. 2015;69(1):1–4.
5. Fritz NE, Cheek FM, Nichols-Larsen DS. Motor-Cognitive Dual-Task Training in Persons With Neurologic Disorders: A Systematic Review. J Neurol Phys Ther. 2015 Jul;39(3):142–53.

6. Ghai S, Ghai I, Schmitz G, Effenberg AO. Effect of rhythmic auditory cueing on parkinsonian gait: A systematic review and meta-analysis. Sci Rep. 2018;8(1).
7. Gordt K, Gerhardy T, Najafi B, Schwenk M. Effects of Wearable Sensor-Based Balance and Gait Training on Balance, Gait, and Functional Performance in Healthy and Patient Populations: A Systematic Review and Meta-Analysis of Randomized Controlled Trials. Gerontology. 2018;64(1):74–89.
8. Harrison SL, Laver KE, Ninnis K, Rowett C, Lannin NA, Crotty M. Effectiveness of external cues to facilitate task performance in people with neurological disorders: a systematic review and meta-analysis. Vol. 41, Disability and Rehabilitation. Taylor and Francis Ltd; 2019. p. 1874–81.
9. Lei C, Sunzi K, Dai F, Liu X, Wang Y, Zhang B, et al. Effects of virtual reality rehabilitation training on gait and balance in patients with Parkinson's disease: A systematic review. PLoS One. 2019; 14(11):e0224819.
10. Mirelman A, Bonato P, Camicioli R, Ellis TD, Giladi N, Hamilton JL, et al. Gait impairments in Parkinson's disease. Lancet Neurol. 2019;18(7):697–708.
11. Perrochon A, Borel B, Istrate D, Compagnat M, Daviet JC. Exercise-based games interventions at home in individuals with a neurological disease: A systematic review and meta-analysis. Vol. 62, Annals of Physical and Rehabilitation Medicine. Elsevier Masson SAS; 2019. p. 366–78.
12. Ramírez-Nieto M, Ortiz-Gutiérrez RM, Cano-de la Cuerda R. Effectiveness of commercial video games in balance and gait treatment in Parkinson's disease | Eficacia de los videojuegos comerciales en el tratamiento del equilibrio y la marcha en la enfermedad de Parkinson. Rehabilitacion. 2018;52(2):114–24.
13. Triegaardt J, Han TS, Sada C, Sharma S, Sharma P. The role of virtual reality on outcomes in rehabilitation of Parkinson's disease: meta-analysis and systematic review in 1031 participants. Neurol Sci. 2020;41(3):529–36.
14. Wang B, Shen M, Wang Y-X, He Z-W, Chi S-Q, Yang Z-H. Effect of virtual reality on balance and gait ability in patients with Parkinson's disease: a systematic review and meta-analysis. Clin Rehabil. 2019 Jul 24;33(7):1130–8.

Not focus on walking speed (n=3)

1. Baram Y, Aharon-Peretz J, Badarny S, Susel Z, Schlesinger I. Closed-loop auditory feedback for the improvement of gait in patients with Parkinson's disease. J Neurol Sci. 2016;363:104–6.
2. Lu C, Twedell E, Elbasher R, McCabe M, MacKinnon CD, Cooper SE. Avoiding virtual obstacles during treadmill gait in Parkinson's disease. Front Aging Neurosci. 2019;11(APR).
3. Yen C-Y, Lin K-H, Hu M-H, Wu R-M, Lu T-W, Lin C-H. Effects of Virtual Reality–Augmented Balance Training on Sensory Organization and Attentional Demand for Postural Control in People With Parkinson Disease: A Randomized Controlled Trial. Phys Ther. 2011 Jun 1;91(6):862–74.

Telerehabilitation (n=1)

1. Shen X, Mak MKY. Balance and Gait Training With Augmented Feedback Improves Balance Confidence in People With Parkinson's Disease: A Randomized Controlled Trial. Neurorehabil Neural Repair. 2014 Jul;28(6):524–35.

No Virtual Reality treatment (n=27)

1. Ashoori A, Eagleman DM, Jankovic J. Effects of auditory rhythm and music on gait disturbances in Parkinson's disease. Front Neurol. 2015;6(NOV).
2. Bondi M, Zeilig G, Bloch A, Fasano A, Plotnik M. Split-arm swinging: The effect of arm swinging manipulation on interlimb coordination during walking. J Neurophysiol. 2017 Aug 4;118(2):1021–33.
3. Castaño Y, Arango J, Navarro A. Spatiotemporal Gait Variables Using Wavelets for an Objective Analysis of Parkinson Disease. Stud Health Technol Inform. 2018;2 49:173–8.
4. Castaño YJ, Navarro A, Arango JD, Muñoz BE, Orozco JL, Valderrama J. Gait and arm swing analysis measurements for patients diagnosed with Parkinson's disease, using digital signal processing and Kinect. In: CEUR Workshop Proceedings. 2018. p. 71–4.
5. Cho C, Kunin M, Kudo K, Osaki Y, Olanow CW, Cohen B, et al. Frequency-velocity mismatch: A fundamental abnormality in parkinsonian gait. J Neurophysiol. 2010;103(3):1478–89.
6. Ehgoetz Martens KA, Pieruccini-Faria F, Almeida QJ. Could Sensory Mechanisms Be a Core Factor That Underlies Freezing of Gait in Parkinson's Disease? PLoS One. 2013;8(5).
7. El-Tamawy MS, Darwish MH, Khallaf ME. Effects of augmented proprioceptive cues on the parameters of gait of individuals with Parkinsons disease. Ann INDIAN Acad Neurol. 2012;15(4):267–72.

8. Ford KJ, Joop A, Memon RA, Wood KH, Ball K, Cutter GR, et al. Pedestrian safety in patients with Parkinson's disease: A case-control study. Mov Disord. 2017 Dec 1;32(12):1748–55.
9. Ghoseiri K, Forogh B, Sanjari MA, Bavi A. The effect of a vibratory lumber orthosis on walking velocity in patients with Parkinson's disease. Prosthet Orthot Int. 2009;33(1):82–8.
10. Ginis P, Heremans E, Ferrari A, Bekkers EMJ, Canning CG, Nieuwboer A. External input for gait in people with Parkinson's disease with and without freezing of gait: One size does not fit all. J Neurol. 2017 Jul 1;264(7):1488–96.
11. Gómez-Jordana LI, Stafford J, Peper CE, Craig CM. Crossing Virtual Doors: A New Method to Study Gait Impairments and Freezing of Gait in Parkinson's Disease. Parkinsons Dis. 2018;2018.
12. Lewis GN. Stride length regulation in Parkinson's disease: the use of extrinsic, visual cues. Brain. 2000 Oct 1;123(10):2077–90.
13. Lin C-C, Wagenaar RC, Young D, Saltzman EL, Ren X, Neargarder S, et al. Effects of Parkinson's disease on optic flow perception for heading direction during navigation. Exp Brain Res [Internet]. 2014 Apr 11;232(4):1343–55.
14. Maidan I, Rosenberg-Katz K, Jacob Y, Giladi N, Hausdorff JM, Mirelman A. Disparate effects of training on brain activation in Parkinson disease. Neurology. 2017 Oct 24;89(17):1804–10.
15. Mazilu S, Blanke U, Hardegger M, Troster G, Gazit E, Dorfman M, et al. GaitAssist: A wearable assistant for gait training and rehabilitation in Parkinson's disease. In: 2014 IEEE International Conference on Pervasive Computing and Communication Workshops, PERCOM WORKSHOPS 2014. IEEE Computer Society; 2014. p. 135–7.
16. Novak P, Novak V. Effect of step-synchronized vibration stimulation of soles on gait in Parkinson's disease: a pilot study. J Neuroeng Rehabil. 2006 Jan;3:9.
17. Oates AR, Van Ooteghem K, Frank JS, Patla AE, Horak FB. Adaptation of gait termination on a slippery surface in Parkinson's disease. Gait Posture. 2013;37(4):516–20.
18. Penko AL, Streicher MC, Koop MM, Dey T, Rosenfeldt AB, Bazyk AS, et al. Dual-task Interference Disrupts Parkinson's Gait Across Multiple Cognitive Domains. Neuroscience [Internet]. 2018 May;3 79:375–82.
19. Pereira VAI, Polastri PF, Simieli L, Rietdyk S, Itikawa Imaizumi LF, Moretto GF, et al. Parkinson's patients delay fixations when circumventing an obstacle and performing a dual cognitive task. Gait Posture.
20. Pineda G, Atehortúa A, Iregui M, García-Arteaga JD, Romero E. Quantifying stimulus-response rehabilitation protocols by auditory feedback in Parkinson's disease gait pattern. In: Proceedings of SPIE - The International Society for Optical Engineering. 2017.
21. Pradhan S. The use of commercially available games for a combined physical and cognitive challenge during exercise for individuals with Parkinson's disease - a case series report. Physiother Theory Pract. 2019 Apr 3;35(4):355–62.
22. Rabin E, Demin A, Pirrotta S, Chen J, Patel H, Bhambri A, et al. Parkinsonian gait ameliorated with a moving handrail, not with a banister. Arch Phys Med Rehabil. 2015 Apr 1;96(4):735–41.
23. Tan D, Pua Y-H, Balakrishnan S, Scully A, Bower KJ, Prakash KM, et al. Automated analysis of gait and modified timed up and go using the Microsoft Kinect in people with Parkinson's disease: associations with physical outcome measures. Med Biol Eng Comput. 2019 Feb 13;57(2):369–77.
24. Ťupa O, Procházka A, Vyšata O, Schätz M, Mareš J, Vališ M, et al. Motion tracking and gait feature estimation for recognising Parkinson's disease using MS Kinect. Biomed Eng Online [Internet]. 2015 Dec 24;14(1):97.
25. Van Hedel H, Waldvogel D, Dietz V. Learning a high-precision locomotor task in patients with Parkinson's disease. Mov Disord. 2006;21(3):406–11.
26. Werner WG, Gentile AM. Improving gait and promoting retention in individuals with Parkinson's disease: a pilot study. J Neurol. 2010 Nov;257(11):1841–7.
27. Werner WG, Gentile AM. Instructional cues and parkinsonian gait: A Pilot Study. Neurol Rep. 2003;27(1):8–14.
